# Iron chelation increases beige fat differentiation and metabolic activity, preventing and treating obesity

**DOI:** 10.1038/s41598-022-04809-8

**Published:** 2022-01-14

**Authors:** Mojgan Nazari, Kenneth W. Ho, Natasha Langley, Kuan M. Cha, Raymond Kodsi, Mawson Wang, D. Ross Laybutt, Kim Cheng, Rebecca A. Stokes, Michael M. Swarbrick, Jenny E. Gunton

**Affiliations:** 1 Centre for Diabetes, Obesity and Endocrinology (CDOE), The Westmead Institute for Medical Research, The University of Sydney, 176 Hawkesbury Rd, Westmead, NSW 2145 Australia; 2 Faculty of Medicine and Health, The University of Sydney, Westmead, Australia; 3 Garvan Institute of Medical Research, Darlinghurst Sydney, Australia; 4 Department of Diabetes and Endocrinology, Westmead Hospital, Sydney, Australia

**Keywords:** Physiology, Endocrinology

## Abstract

Beige and brown fat consume glucose and lipids to produce heat, using uncoupling protein 1 (UCP1). It is thought that full activation of brown adipose tissue (BAT) may increase total daily energy expenditure by 20%. Humans normally have more beige and potentially beige-able fat than brown fat. Strategies to increase beige fat differentiation and activation may be useful for the treatment of obesity and diabetes. Mice were fed chow or high-fat diet (HFD) with or without the iron chelator deferasirox. Animals fed HFD + deferasirox were markedly lighter than their HFD controls with increased energy expenditure (12% increase over 24 h, p < 0.001). Inguinal fat from HFD + deferasirox mice showed increased beige fat quantity with greater *Ucp1* and *Prdm16* expression. Inguinal adipose tissue explants were studied in a Seahorse bioanalyser and energy expenditure was significantly increased. Deferasirox was also effective in established obesity and in ob/ob mice, indicating that intact leptin signalling is not needed for efficacy. These studies identify iron chelation as a strategy to preferentially activate beige fat. Whether activating brown/beige fat is effective in humans is unproven. However, depleting iron to low-normal levels is a potential therapeutic strategy to improve obesity and related metabolic disorders, and human studies may be warranted.

## Introduction

Obesity is the result of excess energy intake compared to energy expenditure over the longer-term. Complications of obesity can include type 2 diabetes, metabolic syndrome, osteoarthritis and many kinds of cancer^1,2^. In the USA more than 50% of adults are overweight or obese and the costs are estimated at $147 billion per year^3^. Methods to decrease weight need to decrease caloric intake, increase activity, increase basal or activated metabolic rate or achieve a combination of these.

A strategy to increase metabolic rate is to increase quantity and activation of beige and brown fat. Brown and beige adipose tissues express the protein UCP1 and consume glucose and lipids to generate heat^4–6^. UCP1 is also used as a marker of their cell phenotype. Humans have relatively limited brown fat, mostly located in the deeper fat layers in the neck^5,7–9^, but may have relatively large quantities of beige fat in the neck and in many other locations as suggested by positron-emission tomography (PET) scanning^10^.

Iron plays an important role in oxidative stress and free radical pathology. Abnormalities of iron status and metabolism are very common and are associated with increased risks of diabetes and obesity^11–13^. In this study the metabolic effects of an oral iron chelator deferasirox (DFS) were examined. The underlying hypothesis was that deferasirox would improve obesity and its metabolic complications by increasing metabolic rate.

## Results

**Figure 1:**
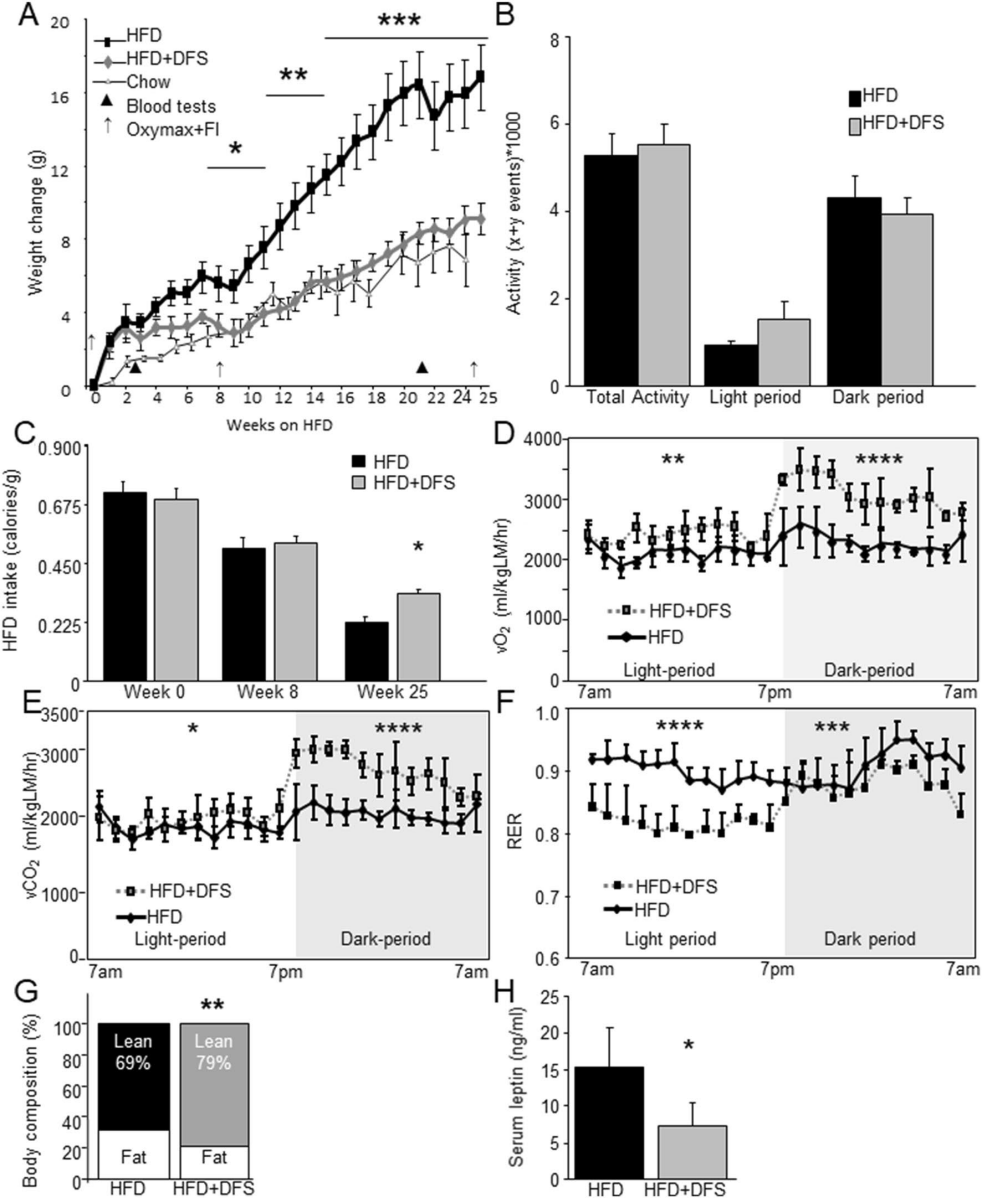
Iron chelation with deferasirox (DFS) prevents high fat diet induced weight gain by increasing energy expenditure. (**A**) Weight gain in mice fed normal chow, high-fat diet (HFD) or HFD + DFS. (**B**) Activity did not differ between HFD and HFD + DFS mice. (**C**) Food intake was similar or higher in mice fed HFD + DFS. (**D**) Oxygen consumption (vO_2_) was higher in mice eating HFD + DFS. (**E**) Carbon dioxide production (vCO_2_) was higher in HFD + DFS mice. (**F**) Respiratory exchange ratio (RER) was lower in HFD + DFS mice. (**G**) Lean mass assessed by dual X-Ray absorptiometry (DEXA) was lower in HFD mass, and fat mass was higher. (**H**) Serum leptin was decreased in HFD + DFS mice. *p < 0.05, **p < 0.01, ***p < 0.001, ****p < 0.0001. Data is shown as mean ± SEM (standard error of the mean) unless otherwise specified.

Eight-week old male C57Bl/6 mice were placed on high-fat diet (HFD, with 45% of calories from fat) plus or minus addition of deferasirox (DFS). Over 25 weeks of diet (Fig. 1A) mice fed HFD + DFS gained substantially less weight than mice fed HFD alone (p < 0.001). By the end of the study, the weight gain from baseline was ~ 40% lower in HFD + DFS mice. Body weight of HFD + DFS mice did not differ from mice which were fed normal chow for the study duration.

Decreased weight gain can be due to decreased food intake, increased exercise, increased energy expenditure or a combination of those factors. Figure 1B shows that mice fed HFD or HFD + DFS did not differ in physical activity levels. Food intake was very similar in the first week, suggesting no issues with palatability (Fig. 1C). By the end of the study, mice fed HFD + DFS ate 30% more food per gram of body weight (p < 0.05). Fecal weight/gram of food eaten was non-significantly lower in HFD + DFS mice (0.14 ± 0.03 versus 0.17 ± 0.03) suggesting that if anything, food absorption was more rather than less efficient with DFS and did not account for lower weight gain.

To determine if whole body metabolism accounted for the differences in body weight, mice were housed separately in metabolic cages. As shown in Fig. 1D, the oxygen consumption (vO_2_) of mice eating HFD + DFS was significantly higher during the light-period, when mice are less active (p < 0.01). There was a proportionally much larger increase in vO_2_ during the night, when mice are active (p < 0.0001). Similar changes in expired carbon dioxide vCO_2_ were seen (Fig. 1E). Overall, this led to highly significant changes in the respiratory exchange ratio (RER, Fig. 1F), with HFD + DFS mice having much lower RER in the daytime. Over the 24 h, HFD + DFS mice showed greater metabolic flexibility, with the lower RER in the day indicating preferential lipid-burning, and the higher RER at night indicating greater consumption of carbohydrates. In contrast, the HFD mice had flatter 24-h profiles, indicating less flexibility and lower lipid consumption.

ANCOVA for repeated measures analysis of the metabolic data with lean-mass as the covariate showed that DFS significantly influenced vO_2_, p = 0.004. Lean mass was also significant (p = 0.032). The effect size of DFS was large (partial η^2^ 0.479) and lean mass was also a substantial contributor (partial η^2^ 0.306).

HFD + DFS mice had a greater proportion of their body weight as lean mass (79 vs 69% in HFD mice, p < 0.01) and a lower percentage as fat mass (Fig. 1G). Serum leptin was ~ 60% lower in HFD + DFS mice (Fig. 1H, p < 0.05) and was significantly correlated with total body mass (r = 0.762, p = 0.001) and inversely correlated with percent lean mass percent lean mass (r − 0.715, p = 0.002).

Because DFS is an iron chelator, a potential side effect is anaemia. No mice were anaemic at the end of the study period. Mean haemoglobin is shown in Supplementary Fig. S1A. Mean corpuscular (red cell) volume (MCV), an indicator of potential iron deficiency, also did not differ (Fig. S1B). Iron studies showed higher serum iron in the HFD + DFS mice (Fig. S1C), consistent with the known increases in serum iron in humans receiving deferasirox. Serum ferritin did not differ (p = 0.6, Fig. S1D). In small numbers, liver function tests did not differ (n = 4, Fig. S1E).

### Fat weight and adipocyte size and fibrosis in mice fed deferasirox

**Figure 2:**
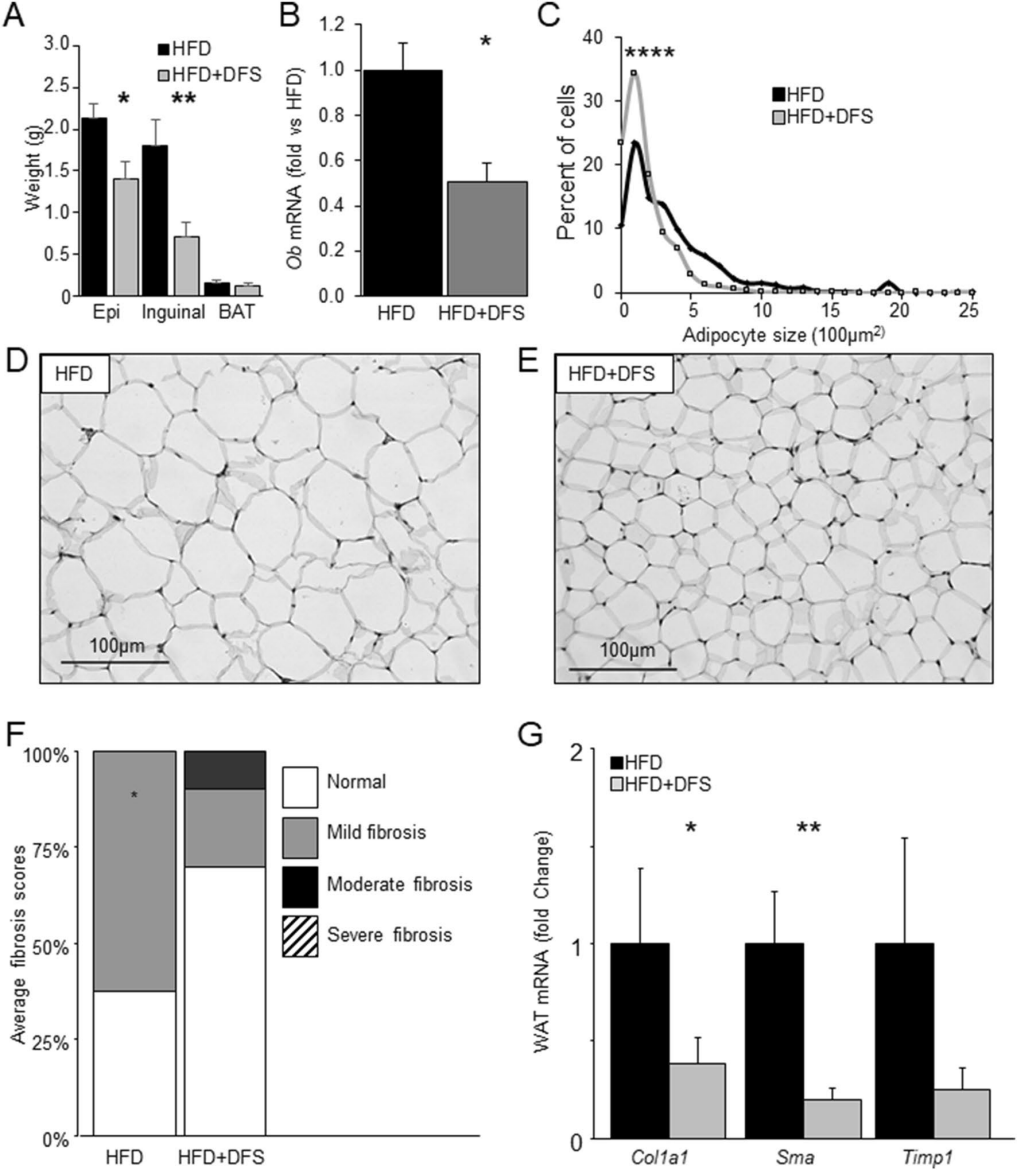
Deferasirox fed mice had lower fat weight and smaller adipocytes. (**A**) Fat pat weights of epigonadal (epi) and inguinal fat were lighter in HFD + DFS mice. Brown adipose tissue (BAT) weight was not significantly different. (**B**) Leptin mRNA (*Ob* mRNA) was lower in fat from mice fed HFD + DFS. (**C**) Adipocytes were smaller mice receiving HFD + DFS. (**D**, **E**) representative histology pictures of fat from HFD and HFD + DFS mice. Scale bars are 100 µm. (**F**) Sirius red stained slides were scored for fibrosis, which was significantly lower in HFD + DFS mice. (**G**) Gene expression of genes associated with fibrosis was lower for Collagen 1a1 (*Col1*), smooth muscle actin (*Sma*) and *Timp1* (metalloproteinase inhibitor 1). *p < 0.05, **p < 0.01. Data is shown as mean ± SEM (standard error of the mean) unless otherwise specified.

At sacrifice the decrease in fat percentage seen with body composition studies was reflected in decreased weights for both inguinal fat (~ 30% decrease) and epigonadal fat (60% decrease, Fig. 2A). There was no significant change interscapular brown adipose tissue weight (BAT). In the fat, and consistent with serum leptin, there was decreased expression of *Ob* mRNA (Fig. 2B). We previously reported significantly better glucose tolerance after 3 weeks of DFS in high-fat-fed mice^14^. Consistent with those findings, mice receiving longer term HFD + DFS had significantly improved fasting glucose (Supplementary Fig. S2A) compared to HFD mice. Glucose tolerance was also significantly improved, Supplementary Fig. S2B. Fasting insulin was reduced by ~ 50% in HFD + DFS mice (Supplementary Fig. S2C p < 0.001). Glucose stimulated insulin secretion (GSIS) was severely impaired in HFD mice (Fig. S2D). HFD mice did not retain first phase insulin release and had impaired second phase insulin secretion, but the normal pattern of insulin secretion was still present in HFD + DFS, although first phase was blunted.

We examined adipocyte size at sacrifice. As shown in Fig. 2C, there was a significant left-shift of cell size in mice fed HFD + DFS compared to mice eating HFD alone. Representative images of the epigonadal fat pads are shown in Fig. 2D and E.

With increasing obesity, adipose tissue experiences increasing relative hypoxia^15–17^. This is associated with increased HIF-1α, and increased adipose depot fibrosis^18–20^. We quantified fat fibrosis using Sirius Red staining and grading of the slides by a researcher masked to study group. No sections had severe fibrosis. As shown in Fig. 2F, HFD + DFS mice had decreased fibrosis of fat with > 60% of slides showing no increase in fibrosis. In Fig. 2G, gene expression of Collagen 1A1 (*Col1*), smooth muscle actin (*Sma*) and *Timp1* were measured by real-time PCR. Consistent with the lower fibrosis, there was significantly lower expression of both Collagen 1A1 and smooth muscle actin in fat from HFD + DFS mice.

### Increased beige fat in mice fed deferasirox

**Figure 3:**
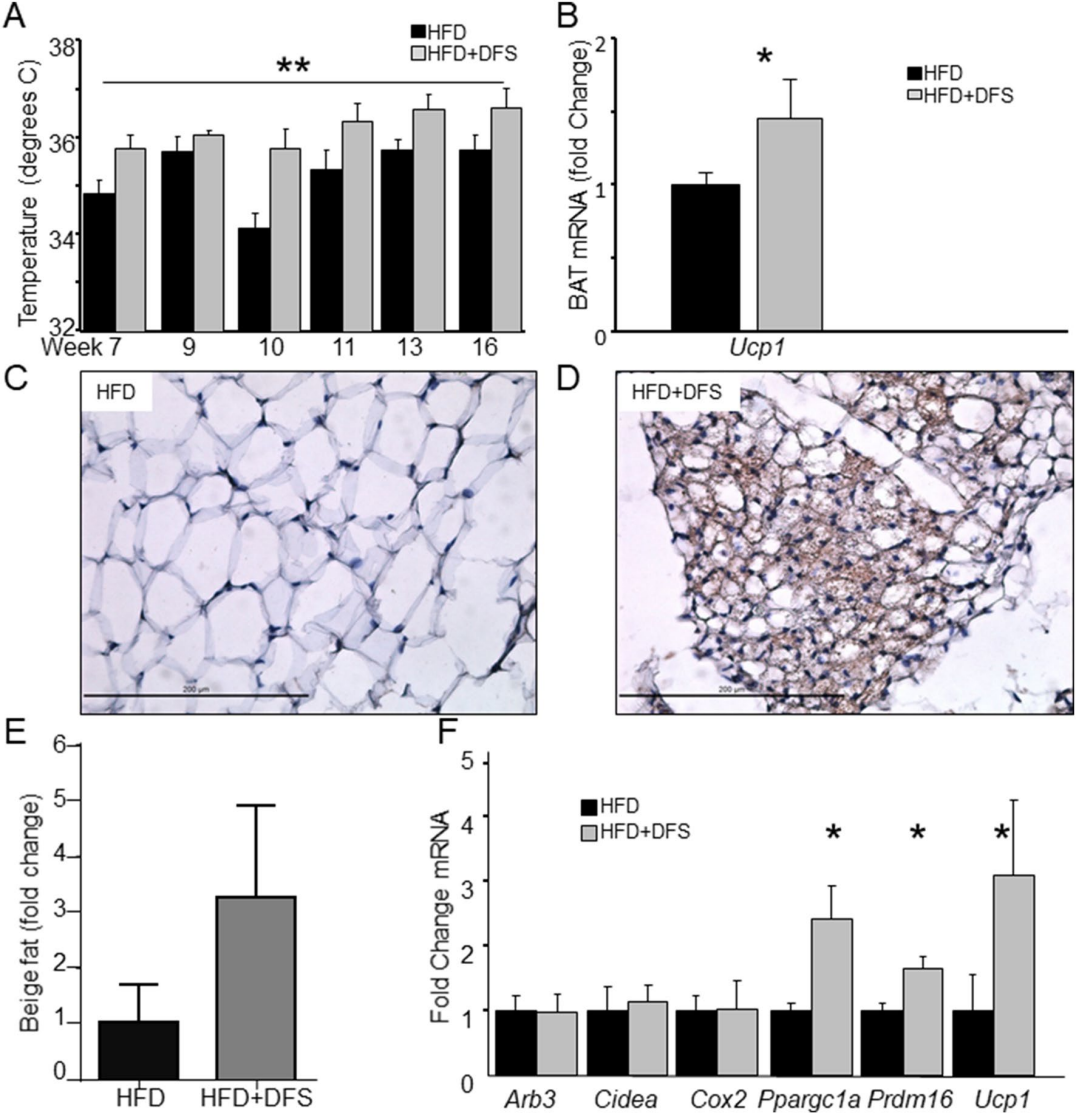
Deferasirox fed mice had increased beige fat. (**A**) Body temperature was higher in HFD + DFS mice (ANOVA for repeated measures). (**B**) In brown adipose tissue (BAT), *Ucp1* mRNA was higher. (**C**, **D**) UCP1 immunostained inguinal fat from HFD-fed and HFD + DFS mice. Scale bars are 200 µm. (**E**) Areas of dense UCP1 staining were quantified. (**F**) Fat from HFD + DFS mice had increased expression of *Ppargc1a, Prdm16* and *Ucp1* mRNAs. *p < 0.05, **p < 0.01. Data is shown as mean ± SEM (standard error of the mean) unless otherwise specified.

Because mice had substantially increased energy expenditure, but no increase in activity, we measured their body temperature. This was significantly increased over time (p < 0.01 by ANOVA for repeated measures, Fig. 3A). No mouse had an abnormally high body temperature at any time tested in either group.

In BAT, *Ucp1* mRNA was 35% higher (p < 0.05) in HFD + DFS mice compared to HFD group (Fig. 3B). However, UCP1 protein in BAT was not changed (data not shown). Together with no increase in BAT weight (Fig. 2A) this suggests that the large increase in energy expenditure was not due to changes in BAT.

Beige fat depots were assessed next. As shown in Fig. 3C and D, which were taken at the same magnification, inguinal fat from mice fed HFD + DFS had patches of markedly browned fat. There was significant staining for UCP1, and many, smaller, multiloculated cells were present. Quantifying these areas showed a > threefold increase in beige fat area in HFD + DFS mice (Fig. 3E). Consistent with the beige fat appearance in inguinal fat, there was a substantial increase in gene expression of *Ucp1* and two other beige/brown fat markers PGC1a (*Ppargc1a)* and *Prdm16* (Fig. 3F).

### Deferasirox is effective after obesity is established

**Figure 4:**
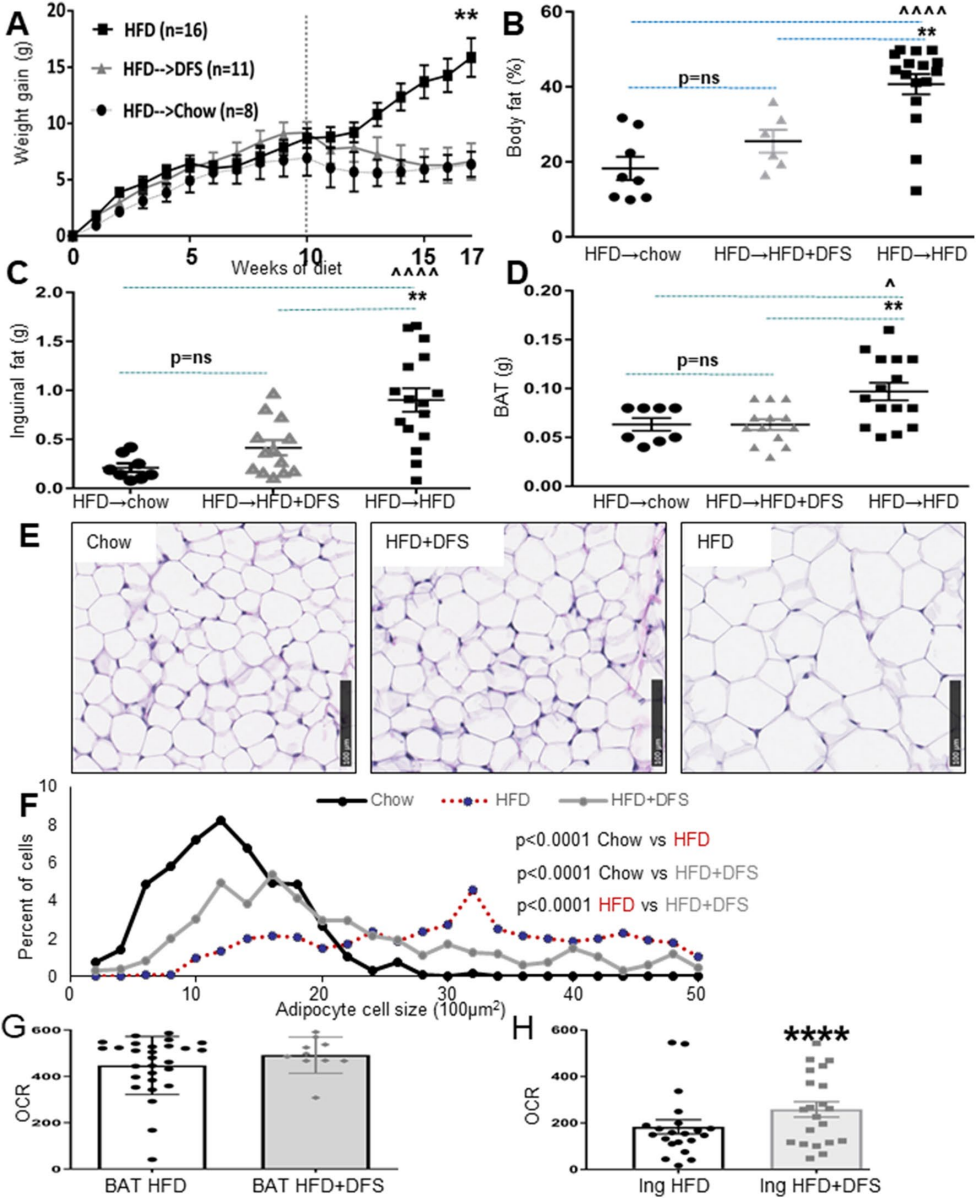
Deferasirox causes weight loss after high-fat diet (HFD) induced weight gain. (**A**) All mice were fed HFD for 10 weeks, then mice were continued on HFD, switched to HFD + DFS or switched to chow. Figure shows weight gain from start of HFD. (**B**) Mice which continued HFD had greater percentage body fat (assessed by DEXA), and (**C**) greater weight of inguinal fat and (**D**) brown adipose tissue (BAT). (**E**) Representative images of fat from chow, HFD + DFS and HFD mice. (**F**) Quantification of fat cell size in mice switched to chow, HFD or HFD + DFS. (**G**) Oxygen consumption rate (OCR) of BAT explants, measured using Seahorse bioanalyzer. (**H**) Oxygen consumption rate of explants of inguinal fat from HFD and HFD + DFS mice. Dots indicate individual values for each mouse. *p < 0.05, **p < 0.01, ***p < 0.001, ****p < 0.0001 DFS + HFD versus HFD mice. ^p < 0.05, ^^p < 0.01, ^^^^p < 0.0001 in HFD versus chow mice. Data is shown as mean ± SEM (standard error of the mean) unless otherwise specified.

Having identified that DFS prevented obesity induction by HFD, we tested whether DFS could treat established obesity. Mice ate HFD for 10 weeks, and then switched to chow, continued on HFD, or switched to HFD + DFS. As shown in Fig. 4A, the mice gained weight on HFD as expected. The chow group lost weight fastest after the diet switch, but by 7 weeks of new diet, HFD + DFS mice no longer differed from mice switched to chow. Chow and HFD + DFS mice weighed markedly less than mice which continued HFD; those mice continued to gain weight with the difference at the end of the study being nearly 10 g of body weight.

Percent body fat was assessed by DEXA (Fig. 4B). Body fat was not significantly different in HFD + DFS switch versus chow mice. Both chow (p < 0.0001) and HFD + DFS mice (p < 0.01) had lower percentage body fat than HFD mice. Figure 4C shows that at sacrifice, inguinal fat pads were significantly larger in HFD mice than the other groups. This was also seen for BAT (Fig. 4D), although histology suggests that the increase in BAT weight in HFD mice was extra lipid, not more classic brown adipocytes. Inguinal adipocyte size was examined (Fig. 4E) and quantified (Fig. 4F). Mice fed HFD + DFS had larger adipocyte size than chow-fed mice, and HFD had the largest adipocyte size.

### Deferasirox increases energy expenditure in inguinal fat explants

To directly assess energy expenditure and overall metabolic function in adipose tissue, oxygen consumption of fat explants was measured in a Seahorse bioanalyser. Consistent with the lack of increase in UCP1 protein in BAT, oxygen consumption rate (OCR) was not increased in BAT (Fig. 4G). Figure 4H shows that in inguinal fat, however, there was 40% higher OCR in HFD + DFS explants compared to HFD, demonstrating greater metabolic activity in inguinal fat (p < 0.0001). As indicated by the individual data points, many of HFD + DFS inguinal explants had OCR similar to BAT (same axis as Fig. 4G) consistent with the observed increases in UCP1.

### Deferasirox is effective in ob/ob mice

Most people with obesity appear to be leptin resistant^21,22^. We tested whether leptin signalling was required for the effects of deferasirox, using the leptin deficient, obese, insulin resistant, ob/ob mouse model^23,24^. Ob/ob mice and their littermate controls were randomized to chow + DFS or chow-fed groups (cages were randomised by coin-toss). Control mice also received powdered chow to equalise the effort required to chew food, and DFS mice received powdered chow mixed with DFS.

**Figure 5:**
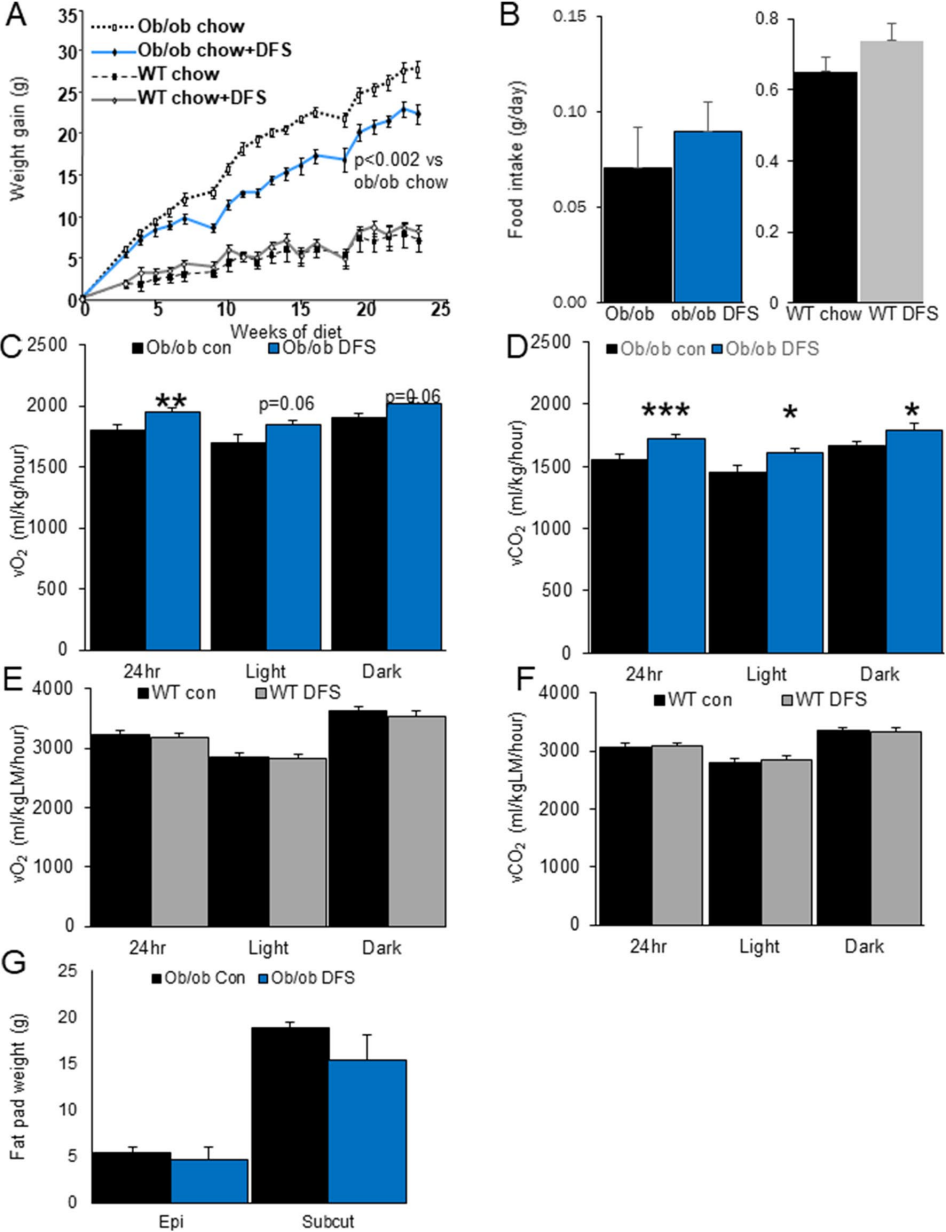
Deferasirox decreases weight gain in ob/ob leptin deficient mice. (**A**) DFS + chow resulted in lower weight gain in ob/ob mice compared to mice eating chow. (**B**) Food intake was not decreased in ob/ob mice eating DFS. (**C**) Oxygen consumption (vO_2_) was higher in ob/ob chow + DFS mice. (**D**) Carbon dioxide production (vCO_2_) was also higher in ob/ob chow + DFS mice. (**E**) No differences in vO_2_ were seen in chow versus chow + DFS fed wild type littermates. (**F**) No differences in vCO_2_ were seen in chow versus chow + DFS fed wild type littermates. (**G**) Fat pad weights of epigonadal and subcutaneous inguinal fat of ob/ob mice at sacrifice. *p < 0.05, **p < 0.01, ***p < 0.001. Data is shown as mean ± SEM (standard error of the mean) unless otherwise specified.

Figure 5A shows that ob/ob mice fed chow gained 24% more weight compared to chow + DFS ob/ob mice. This became significant 7 weeks after starting the diet. In contrast, wild-type littermates receiving chow ± DFS did not have altered weight gain (Fig. 5A) compared to chow-fed controls.

Ob/ob mice on DFS did not eat less (Fig. 5B). However, ob/ob mice fed chow + DFS had higher vO_2_ (12.5% increase over 24 h, p < 0.0001, Fig. 5C) and vCO_2_ (15% higher, p < 0.0001, Fig. 5D). WT mice fed chow and chow + DFS did not differ in either vO_2_ (Fig. 5E) or vCO_2_ (Fig. 5F). At sacrifice, there was wide variability in tissue weights in ob/ob mice, and fat weights did not differ significantly (Fig. 5G).

## Discussion

Increasing thermogenesis with deferasirox prevents obesity in mice fed HFD and is successful at treating obesity previously induced by HFD. This is achieved without significant drop in haemoglobin and iron status is lower-normal, not deficient. Iron chelation also reduces weight gain in leptin-deficient ob/ob mice fed normal chow. This indicates that normal leptin signalling is not required for beneficial effects of decreasing iron load.

Decreased weight must be due to lower caloric intake, increased energy expenditure, or both. Mice fed deferasirox did not eat less in any experiment, including in the first week after diet change, indicating that palatability was not affected. In the later part of the studies, iron chelator-fed mice tend to eat more, while maintaining lower weight. Iron chelation leads to a substantial increase in oxygen consumption, accompanied by increased carbon dioxide production. Night and daytime oxygen consumption are increased, with a larger increase in the dark-hours when mice are usually more active. There is a preservation of the normal pattern of change in respiratory exchange ratio (RER) in mice fed HFD + DFS compared to a flattened 24-h profile in mice eating HFD. This is consistent with appropriate shifting between burning of lipids and carbohydrates for fuel with iron chelation.

The mechanism of the increase in energy consumption was studied. There is no significant change in weight of interscapular brown adipose tissue in mice receiving deferasirox. Surprisingly, although there is a statistically significant, small increase in *Ucp1* mRNA in BAT, there is no significant change in UCP1 protein on Western blotting. In addition, explants of BAT showed no difference in oxygen consumption in mice easting HFD + deferasirox compared to mice fed HFD alone. The lack of increased oxygen consumption is in keeping with the lack of change in UCP1 protein in BAT^25^. These findings suggest that BAT is not an important contributor to the anti-obesity effects seen with iron chelation.

Next, we examined inguinal fat as this is a classically brown-able or beige fat depot in mice. There is a > threefold increase in small, multiloculated, UCP1 positive cells in inguinal fat in mice receiving the iron chelator. This is accompanied by significant increases in expression of *Ucp1* and other classic beige/brown fat genes including *Ppargc1a* which encodes PGC1α. We assessed energy expenditure in the inguinal fat depots using fat explants. Inguinal fat removed from mice fed deferasirox has significantly higher oxygen consumption, with a 40% increase. Notably, 7 of 21 explants of inguinal fat from HFD + DFS mice have high oxygen consumption rates which are similar to those measured in interscapular brown fat.

Additional evidence to suggest that the effects are driven by beige fat are seen in the weight curves, with little to no effect for at least 2 weeks. If BAT were the major driver of the anti-obesity effect, since it is present at the start of the experiment, the treatment should cause weight deviation from the outset.

We are not aware of other agents which preferentially activate beige fat without having significant effects on brown adipose tissue. Although it is now recognised that there is heterogeneity in brown adipose tissue cells^26^, the BAT explant studies do not show any increase in oxygen consumption from mice treated with DFS. PRDM16 ablation is known to cause a preferential deletion of beige fat^27^. The opposite effects are seen with PRDM16 overexpression; increased beige fat and energy expenditure and lower weight gain^28^. We found increased PRDM16 in inguinal adipose tissue from mice fed HFD + DFS.

Usually beige fat plays a much smaller role in whole body metabolism in mice^25^, so it is interesting to note that the oxygen consumption in the beige depots were within the range of BAT in some of the explants from HFD + DFS mice. Because of smaller total volume and UCP1 protein content, usually beige fat does not consume comparable amounts of energy to BAT^25^. However, with some explants showing BAT-like oxygen consumption and ~ 6–8-fold more inguinal fat than BAT in the HFD + DFS mice, increased beige fat energy expenditure in this model could potentially consume more energy than the activated BAT.

Activating brown and beige fats increases insulin sensitivity in people^29^ and increasing activity with iron chelation may therefore improve glucose tolerance. In human adults, RNA-sequencing of brown fat depots shows a more beige-like phenotype rather than classic brown gene signatures^7,8^. The inter-scapular brown fat in infant humans has a classic brown transcriptional signature, and in some studies deep-layer human neck fat has a brown-like phenotype^30^. But overall, humans have more beige-inducible fat than true brown fat, so the preferential effect on beige fat may give improved outcomes.

Worldwide, obesity rates are climbing in most populations. The high rates of obesity are associated with overnutrition and decreased average exercise levels. There are strong epidemiological associations between iron intake, and particularly heme iron intake, and risk of obesity and diabetes^31–33^. Two studies suggest that insulin induces brown fat iron uptake, and that iron increase in turn causes adipose tissue remodelling and adipose tissue insulin resistance^34,35^.

Taken together, our studies provide a possible mechanistic explanation for the links between iron status and obesity and diabetes. We suggest that lowering iron status to the low-normal range in people with high iron status may improve their metabolic rate. Increasing metabolic rate by activating beige fat increases calorie consumption and activated beige fat clears glucose and lipids. This may be a useful strategy to improve metabolism and possibly weight in people with diabetes and obesity related dyslipidemia.

## Materials and methods

### Animals and study design

Eight-week old C57BL/6 male mice (n = 30) were purchased from Australian Bio Resources (Moss Vale, NSW) and delivered to the Biological Services Facility at the Westmead Institute for Medical Research (WIMR). Mice (maximum 5 per cage) were housed in a room at constant temperature (21 °C) with 12 h light/dark cycle. Mice were provided with ample bedding material and plastic ‘houses’.

Food was provided ad libitum unless specified (e.g. fasting for GTT). At study completion, mice were euthanized by ketamine + xylaxine followed by cervical dislocation. Inguinal, subcutaneous, and interscapular brown fat were excised, weighed, and stored in formalin or liquid nitrogen. All procedures conducted on mice were in accordance with the Guide for the care and use of laboratory animals and approved by Western Sydney Local Health District Animal Ethics Committee under AEC protocol number 4222 and Garvan Animal Ethics Committee number #08/28.

### Mouse diets

High fat diet (HFD) containing 45% of calories from fat was prepared according to the Rodent research diet number D1245. To make HFD + DFS (Novartis), 750 mg DFS was ground by mortar and pestle and mixed thoroughly with the dry ingredients. Based on average food consumed, the dose of deferasirox was ~ 20 mg/kg/day. Cages were randomised using the random number generating function in excel where there were > 2 groups, or by coin toss where there were 2 groups. No mice were excluded from the studies.

### Glucose and insulin tolerance tests (GTT and ITT), serum insulin, leptin, iron studies

GTTs were performed as previously reported^36^ in conscious un-restrained mice following a 4-h fast. When the glucometer showed “HI”, glucose was recorded as 30 mmol/L. Mice were fasted for 4 h for ITTs and given actrapid, (Torrent Pharmaceuticals, India) diluted 1:2000 in phosphate buffered saline with 1% bovine serum albumin (BSA), at 0.625 U/kg body weight. Serum insulin was measured as previously reported, by ELISA (Crystal Chem)^37^. Serum leptin was measured in fasting animals by ELISA (Crystal Chem). Iron studies were measured by the pathology laboratory of St Vincent’s Hospital, Sydney.

### Dual energy X-ray absorptiometry (DEXA) scanning

Body composition was assessed using a dual energy x-ray absorptiometry (DEXA), (GE Lunar PIXI-mus, Madison, USA) housed in the Kids Research Institute, Westmead Children’s hospital, Westmead. Mice were anesthetised using isoflurane Scanning was performed as per manufacturer’s protocol. Mice were monitored until full recovery from the anaesthetic.

### Indirect calorimetry

Metabolic measurement was performed using Promethion or Oxymax metabolic cages. Measurements were taken with 12-h light/dark cycle. Mice were housed individually, and acclimatized for 1 day prior to data collection. Cages were fitted with sensors to measure food intake, total activity, difference in oxygen (consumption used to calculate VO_2_) and carbon dioxide (production used to calculate VCO_2_), and heat production or energy expenditure (EE). Oxygen consumption (VO_2_) and carbon dioxide production (VCO_2_) were measured in ml/kg/min. Oxymax cages did not measure food intake. Food intake was measured by putting individual mice in clean cages with a pre-weighed amount of food and weighing the food remaining after 24 h.

Analysis of vO2 by ANCOVA with lean mass as the covariate was done with SPSS version 25.

### Gene expression analysis by real-time PCR

Tissue samples were collected freshly from animals and frozen immediately in liquid nitrogen. RNA was extracted using TRI Reagent (Sigma-Aldrich), or an RNeasy kit (Qiagen) according to the manufacturer’s protocol. Complementary-DNA (cDNA) was synthesised using the Maxima First Strand cDNA Synthesis Kit for RT-qPCR (Thermo-Fischer Scientific) following the manufacturer’s instructions. Real-time PCR was performed using SYBR Green and 18S as an internal loading control. The samples were run in a 384 well CFX384 Touch Real-Time PCR Detection System (Bio-Rad). Primers were used in this study are shown in Table 1. Relative gene expression was calculated using the ^∆∆^Ct method.

**Table 1:** Primer sequences used for qPCR.

Target	Forward primer	Reverse primer
*Cyclophilin*	TGGACCAAACACAAACGGTTCC	ACATTGCGAGCAGATGGGGTAG
*Tbp*	TATCACTCCTGCCACACCAG	ATGATGACTGCAGCAAATCG
*Ucp1*	GCATTCAGAGGCAAATCAGC	GCCACACCTCCAGTCATTAAG
*Cidea*	CATACATGCTCCGAGTACTGG	CATCCCACAGCCTATAACAGAG
*Pgc1α*	CCCTGCCATTGTTAAGACC	TGCTGCTGTTCCTGTTTTC
*Ppargc1α*	GCGTACGGCAATGGCTTTAT	GAACGGCTTCCTCAGGTTCTT
*Prdm16*	CAGCACGGTGAAGCCATTC	GCGTGCATCCGCTTGTG
*Leptin*	AGGATCTGAGGGGTGATGTG	AGGTGACCAAGGTGGCATAG

### Histological analysis

Epigonadal fat, inguinal fat, and intra-scapular brown fat tissues were harvested and fixed in 10% formalin. Formalin-fixed tissue was embedded in paraffin and 5 μM sections were cut.

For green fluorescent protein (GFP) and UCP1 immunofluorescence staining, antigen retrieval was performed by heating the sample in a microwave for 14 min at full power in citrate buffer [10 mmol/L citric acid, pH 6.0, 0.05% Tween20 (Sigma, St Louis, MO, USA)]. Slides were blocked in Protein Block Serum-Free Solution (Dako, Carpinteria, CA, USA) for 30 min at room temperature. They were then washed in DPBS (Lonza) and incubated with primary antibodies diluted in DAKO Antibody Diluent, overnight at 4 °C (rabbit anti-eGFP, Abcam #ab290, 1:1000; and rabbit anti-UCP1, Abcam #ab10983, 1:250). The next day, after 3 washes with DPBS (Lonza), sections were incubated in secondary antibody (Goat anti-rabbit IgG H&L #ab150077, 1:750), and DAPI (DAPI, #62248, Thermo-Fisher Scientific, 1:500 (1 mg/mL), diluted in DAKO Antibody Diluent, for one hour at room temperature. Then slides were washed three times with PBS and cover slips were mounted using Fluoromount aqueous mounting media (Sigma, USA). Images were taken using the Olympus BX53 microscope and XCite 120Q Fluorescence Light Source.

UCP1 + adipose tissue area was calculated as a percentage of the total section, in at least 5 separated sections, multiplied by total depot weight.

### Seahorse analyzer: O_2_ consumption analysis

Fat explants were collected and 10 mg pieces were placed in each well of a Seahorse 24-well plate (Aligent, Santa Clara, CA) with DMEM media with 5 mM glucose and 1% albumin. An XF24 extracellular flux analyser measured oxygen consumption rate (OCR) and extracellular acidification rate (ECAR).

### Statistics

Statistics were calculated using Excel, GraphPad Prism version 7, or for multiple comparisons ANCOVA, by SPSS version 25. Where multiple comparisons were made, p-values were corrected using the Bonferroni technique. A p-value of < 0.05 was considered statistically significant. Unless specified otherwise, figures show mean ± standard error of the mean (SEM).

### Ethical approval

The study is reported in accordance with ARRIVE guidelines.

## Supplementary Information

Supplementary Figure S1.

Supplementary Figure S2.
