# Supplementary figures and images for: Iron chelation increases beige fat differentiation and metabolic activity, preventing and treating obesity
Source: Sci Rep. 2022 Jan 14;12:776. doi: 10.1038/s41598-022-04809-8 (PMC8760280; doi:10.1038/s41598-022-04809-8)

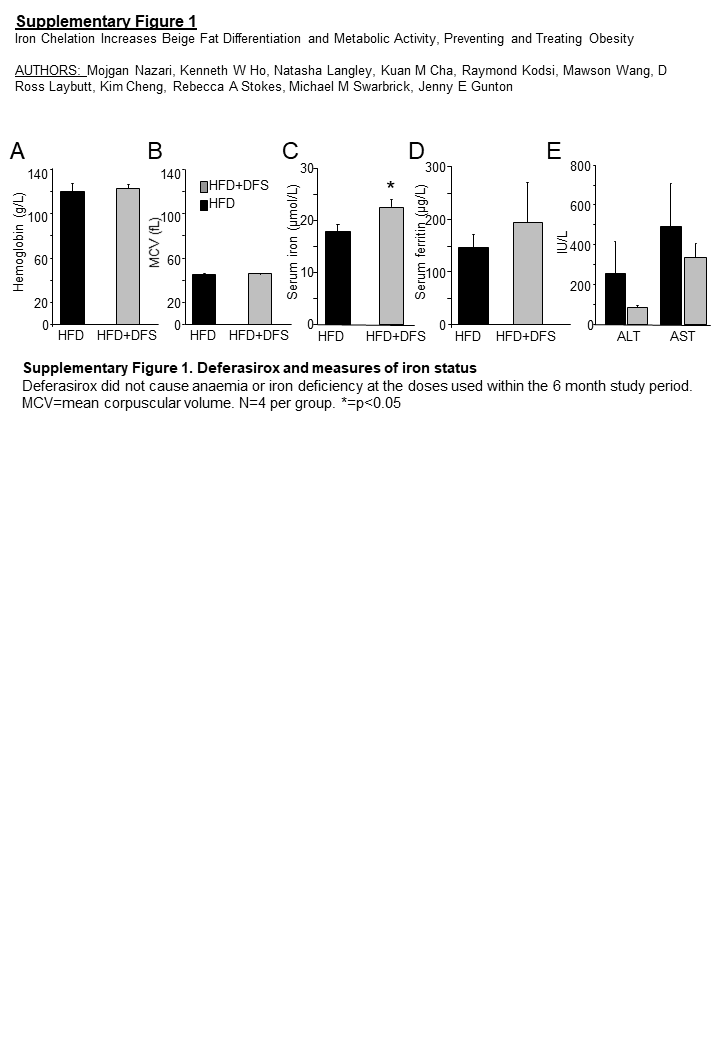

Supplement: Supplementary file 1 — Supplementary Figure S1. [file 41598_2022_4809_MOESM1_ESM.tif]

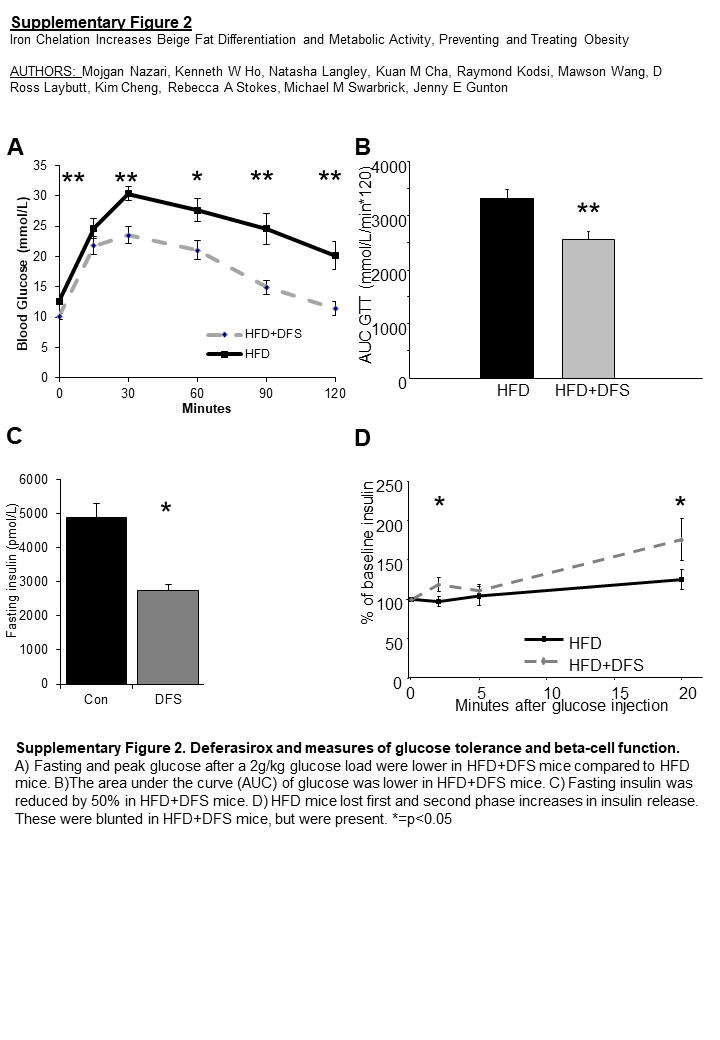

Supplement: Supplementary file 2 — Supplementary Figure S2. [file 41598_2022_4809_MOESM2_ESM.tif]
